# MEMBER CHECKING GERONTOLOGY: THE CASE OF RETIREMENT

**DOI:** 10.1093/geroni/igac059.2503

**Published:** 2022-12-20

**Authors:** David Ekerdt

**Affiliations:** University of Kansas, Lawrence, Kansas, United States

## Abstract

Gerontology has long been a public-facing field with an applied focus. As such, the credibility of gerontology’s conclusions and guidance about aging are crucial, our advice having relevance and impact in proportion to its popular resonance. In 2021 I authored an article for a large-circulation newspaper that generated over 500 reader replies, creating an opportunity for member checking of a kind. The article reported my personal experience of having retired—what I expected and what was a surprise. All of my observations about emotions and lifestyle, while my own, were nonetheless grounded in the research literature. Public comments on the article came from a readership that skews male and highly educated, i.e., people like myself. Many comments affirmed my observations (e.g., about time use, awareness of finitude) as experiences we had in common. Some comments disputed my authority, as an academic, to say anything valid about the “real world.” Opinion split on the value of continued work: it gives life meaning, it invites corrosive stress. Likewise, some retirees endorsed surrender to leisure while others urged engagement. One research takeaway: with no standard way to be retired or regard it, the quality of retired life remains a measurement challenge. Another takeaway: Retirees with partners commonly describe experience in the first-personal plural (we, us), suggesting that dyads are often apt units of analysis for retirement studies. This is but one case study, but it indicates that we must continually assess whether gerontology’s knowledge is valid and whether the public is grateful for it.

